# Advancing Trauma Systems in the United States: Bridging Disparities Through State‐Level Legislation and a Health Systems Approach

**DOI:** 10.1111/1475-6773.70073

**Published:** 2025-12-01

**Authors:** Bilal Irfan, Zain Hashmi, Tatiana Ramos, Molly Jarman

**Affiliations:** ^1^ Center for Bioethics Harvard Medical School Boston Massachusetts USA; ^2^ Center for Surgery and Public Health, Brigham and Women's Hospital Boston Massachusetts USA; ^3^ Department of Neurology University of Michigan Medical School Ann Arbor Michigan USA; ^4^ Department of Epidemiology University of Michigan School of Public Health Ann Arbor Michigan USA; ^5^ Department of Surgery The University of Alabama at Birmingham Birmingham Alabama USA

## 1 Introduction

Traumatic injury is a leading cause of death and disability across all age groups in the United States, yet major gaps persist in the provision and coordination of trauma care [1]. The National Safety Council estimates an economic burden of US $1.3 trillion per year in direct medical costs, work‐loss, and quality‐of‐life decrements, while CDC modeling places the broader societal cost of injury at US $4.2 trillion [2, 3].

Survival after severe injury can depend on where the patient is located. County‐level analyses show the risk of prehospital trauma death is 25% higher in small fringe‐metropolitan counties and 69% higher in rural non‐core counties than in large metropolitan cores [4], and trauma patients from rural communities are 14% more likely to die from their injuries compared to urban residents [5]. Rural communities already face recorded disparities in access to care and poorer health outcomes; for example, the natural‐cause mortality (NCM), defined as stemming from disease‐related deaths, for the working age population (25–54 years) in rural areas is 43% higher than their urban/metropolitan counterparts [6]. National estimates indicate that under‐triage remains widespread: almost one‐half of trauma patients who ultimately die in the emergency department arrive at non‐trauma centers, including 86% of rural cases and 36% of urban cases, showcasing some of the stark geographic gaps in access to definitive care [7]. Contemporary registry data corroborate the problem, showing that one in five injured patients whose injuries truly warrant a full trauma team activation still only receive a limited response, with under‐triage rates differing across trauma center levels and patient demographics [8].

Nearly 20 years since trauma centers were established as the best source of care for critically injured patients [9], and 10 years after the National Academies of Science, Engineering, and Medicine called for a robust national trauma system, disparities in trauma outcomes continue to plague many states and vulnerable populations [10]. These disparities are exacerbated by uneven legislation, variable funding for local emergency medical services (EMS), and persistently high rates of under‐ and over‐triage [11, 12]. A health systems approach, combined with responsive legislation at all levels of government, is essential to address these deficiencies. State‐level legislation, in particular, has the potential to play an important role in complementing federal legislation, especially at times when federal priorities may be structurally misaligned with the needs of different regions. In this commentary, we (i) synthesize where US trauma systems underperform, (ii) situate those gaps against international experience and US trends, and (iii) propose concrete, state‐led legislative levers, paired with federal catalysts and public reporting, to reduce under‐ and over‐triage, strengthen the EMS workforce, integrate data, and extend the trauma continuum into rehabilitation.

## 2 Legislative Fragmentation and Its Consequences

Many states now enshrine trauma center designation in statute, but the substance of those statutes diverges sharply (Table 1). While some define levels of care by American College of Surgeons verification criteria, others designate using seemingly arbitrarily defined levels or leave designation to individual hospitals. Thirty‐two states provide no funding to designated trauma hospitals for readiness or uncompensated care costs [13]. In states where fiscal support is weak, the predictable result is a patchwork of trauma care systems in which Level I centers cluster around affluent urban corridors while entire rural regions may lack 24‐h surgical coverage.

**TABLE 1:** Current weaknesses and challenges in US trauma systems.

Domain	Rationale	Consequences
Preparedness	Incomplete surge coordination across agencies and states	Strained responses to MCIs, wildfires, and hurricanes, and other humanitarian emergencies
Triage and transfer	High under‐triage in older adults; inconsistent re‐triage rules; post‐pandemic IHT acceptance declines	Avoidable mortality/disability; long ED boarding in sending facilities; inequities by residence/insurance
ED operations	Systemic boarding/crowding not addressed by trauma policy	Under‐recognition of time‐critical injury; throughput delays
Rehab and long‐term outcomes	Weak transitions; limited coverage for post‐injury home‐based services	Lost functional recovery; higher readmissions/costs
Governance and finance	Fragmented statutes; many states lack stable trauma/EMS funding; “essential” EMS without enforceable standards	Uneven coverage; rural readiness gaps; closure risk for low‐margin hospitals/EMS
Designation and regionalization	Variable criteria; clustering of Level I centers in urban corridors	Dilution/competition in cities; access deserts in rural regions
Data and measurement	Non‐harmonized registries; limited multi‐payer linkage; little functional outcomes tracking	Policy driven by in‐hospital mortality alone; poor accountability
Workforce	EMS wage/benefit disparities; training/professional‐development gaps	Turnover, thin clinical judgment capacity in rural areas

Although a growing number of states now characterize emergency medical services as “essential,” the statutory meaning of that term remains contested, which produces material consequences for financing and oversight. A current National Conference of State Legislatures analysis finds that, as of June 27, 2025, at least 21 states and the District of Columbia have enacted laws explicitly defining EMS as essential, yet the accompanying duties, funding guarantees, and minimum service standards differ markedly across jurisdictions [14]. Some states provide only a declaration without earmarked revenue or enforceable readiness requirements, while others avoid the “essential” label but impose concrete planning or service mandates and, in some cases, authorize dedicated funding streams or taxing authority. Iowa, for example, permits counties to declare EMS essential and to secure voter‐approved surtaxes or property levies to support implementation; North Carolina requires every county to ensure access to EMS and empowers counties to regulate franchise numbers, service areas, and rates; and California compels local agencies to submit comprehensive EMS plans addressing manpower, communications, transport, data collection, and disaster response [14]. These heterogeneities complicate regionalization and lead to the paradox that a state may “count” as essential in name without stable financing, while another delivers enforceable coverage standards without the label. A coherent state‐level approach should therefore define the obligation, specify minimum coverage and response benchmarks, and tie the designation to predictable funding and transparent reporting.

Regional trauma care is predicated on timely patient access to appropriate resources [15]. However, rural areas frequently have limited prehospital resources, protracted transport times, and fewer trauma centers. Existing needs‐based assessment tools sometimes recommend adding centers in these sparsely populated areas, but financial and workforce constraints often persist [16]. In contrast, some large urban areas may harbor an overabundance of trauma centers—raising concerns about dilution of expertise and competition rather than cooperation. Recognizing that one size does not fit all, system‐wide metrics and a unified yet flexible national research and policy framework are required to improve resource allocation.

Data silos reinforce these disparities. Unlike national stroke and myocardial‐infarction registries, most state trauma registries are housed in departments of transportation or public safety, collect incompatible variables, and either prohibit or price‐gate data sharing across borders. Researchers attempting to link prehospital run sheets with inpatient outcomes often negotiate separate data use agreements for each county or state, a logistical barrier that has stymied large‐scale effectiveness studies. The absence of routine linkage to rehabilitation or physician‐fee datasets means that policymakers may still judge system performance by in‐hospital mortality alone, ignoring long‐term disability that accounts for injury‐related economic loss.

State legislation plays an important role in shaping trauma system funding, data collection, and oversight. States differ widely in their regulatory definitions, EMS oversight agencies, and processes for designating trauma centers [17]. This generates confusion for patients and EMS providers, and perpetuates misalignment of local, state, and federal policies. Addressing these gaps requires coordinated legislative action targeting multiple levels of a health system. Stable funding streams for trauma system infrastructure, robust data repositories, EMS workforce development, and evidence‐based triage guidelines are needed. Legislation should incorporate accountability measures where states commit to transparent monitoring of metrics such as under‐triage, timely transport of severely injured patients, and interfacility transfer patterns.

Disparities among older adults are especially concerning as panel studies reveal that older patients remain disproportionately under‐triaged, sometimes because standard physiologic cut‐points (e.g., blood pressure, Glasgow Coma Scale thresholds) lack sensitivity for frail or anticoagulated older adults [18]. A national Medicare study reports that almost half (46%) of severely injured adults aged 65 years or older are under‐triaged to non‐trauma centers [19]. Field triage guidelines, as recommended by the CDC, now contain some modifications for older adults, but real‐world under‐triage rates remain substantial [11]. Legislating mandatory statewide data integration and adoption of age‐specific triage criteria could help. Yet many trauma surgeons caution that simply lowering the thresholds risks swinging the pendulum toward over‐triage: broad age‐based triggers can relocate older people with low‐severity injuries away from family supports, lengthen transport times, and inflate costs without a proven survival benefit. An emerging view therefore is not to send every older adult to a Level I center, but rather to adopt nuanced, age‐specific triage algorithms that incorporate frailty indices, anticoagulation status, and mechanism of injury. A 100% catch rate for major injury remains the goal, but it must be balanced against the harms of unnecessary transfer. Legislative frameworks could encourage telemedicine and remote consultation to support rural EMS providers. Many states' usage of teletrauma has been associated with rural location, and North Dakota, South Dakota, and Arkansas all serve as examples of high rates of reported teletrauma usage [20]. By focusing legislative priorities on coverage for time‐critical procedures and trauma center readiness, policymakers could tangibly reduce disparities for older and rural populations. Several states offer operational models to consider looking at: North Carolina's county‐level EMS obligations with franchising authority (though it is being partly phased out), and California's statewide planning, trauma registry, and annual EMS performance reporting, attempt to translate statutory intent into enforceable coverage standards and transparent metrics.

Workforce capacity is another important limiting factor. Preliminary interviews our group conducted with regional stakeholders point to certain themes: chronic EMS staffing shortages, especially in rural regions, driven by limited professional‐development opportunities and stark benefit disparities. Unlike fire and police personnel who can retire after 20 years, paramedics in many states must serve 30 years to receive comparable pensions, and salaries seldom keep pace with those of other public‐safety roles. Turnover erodes local expertise just as more sophisticated geriatric triage tools demand higher clinical judgment. Legislation that couples trauma‐system funding to competitive EMS wages, tuition assistance, and paid continuing‐education stipends, while harmonizing retirement benefits with other first‐responder services, would strengthen the human infrastructure needed to deliver equitable trauma care. Providers are also often compelled to make triage decisions amid high rates of ED boarding and crowding (especially since the COVID‐19 pandemic), which can degrade the nature of clinical encounters and can be associated with delays and error, especially for patients who self‐present rather than arrive by EMS [21, 22]. Incorporating ED boarding time into trauma system metrics, and funding hospital‐wide flow solutions, not just ED operations, should be part of the legislative package.

A second area of legislative focus lies in post‐injury disability and rehabilitation. Too often trauma care policy centers on in‐hospital mortality, ignoring functional recovery, mental health, and long‐term impacts on quality of life [23]. Data linkages that follow patients after discharge (EMS through acute care and rehabilitation) remain incomplete in many systems. Funding for integrated data systems and mandatory reporting, akin to state‐level databases used in stroke or ST‐elevation myocardial infarction (STEMI) registries, would help measure outcomes beyond survival. This approach would align with the National Academies of Sciences, Engineering, and Medicine 2016 recommendation to build a national trauma learning health system spanning prehospital, hospital, and rehabilitation phases [9].

Mass casualty incidents showcase the limitations found in current trauma systems. Events ranging from natural disasters to mass shootings strain capacity and reveal fragmented or inadequate planning. Legislative initiatives that formalize and fund robust regional preparedness, with the capacity to rapidly shift resources or coordinate multi‐agency responses, could be transformative [24]. These initiatives should include consensus‐based operational plans, scalable telemedicine platforms, and refined triage strategies for large‐scale incidents.

## 3 Evidence‐Based Legislative Solutions: State Exemplars and Early Failures

Dedicated, performance‐tied financing offers an empirical signal of success. Texas channels revenue from traffic‐violation surcharges into the Designated Trauma Facility and EMS Account (Fund 5111). In July 2024, the Department of State Health Services disbursed US $8.74 million from this account to 290 eligible designated trauma hospitals under the FY 2024 Uncompensated Trauma Care allocation, using the statutory formula that awards 15% of available funds equally and 85% in proportion to each hospital's reported unreimbursed trauma charges [25]. The Maryland Trauma Physician Services Fund, financed by a US $5 surcharge on every motor‐vehicle registration and renewal, disbursed about US $11.6 million in FY 2023, the great majority of which went to on‐call and standby stipends, with the balance covering uncompensated‐care and Medicaid supplemental payments to trauma physicians [26]. Attempts to replicate the model elsewhere sometimes founder on the details of the design and its implementation; they may fail if the state legislature strips accountability clauses, if eligibility and distribution formulas are not tied to readiness or unreimbursed trauma care, and might prompt rural legislators to question value for money.

Pre‐hospital innovation can thrive when statutes uncouple revenue from transport. In Arizona, that decoupling now rests on two complementary mechanisms. First, the state's Treat and Refer Recognition Program authorizes certified EMS agencies to bill Medicaid about US $252 (code A0998 with the “CG” modifier) when a 911 call ends in treatment‐in‐place or a telehealth‐guided hand‐off rather than transport to an emergency department [27]. Second, Arizona's Medicaid agency (AHCCCS) joined the CMS Emergency Triage, Treat, and Transport (ET3) model in October 2021, extending equivalent reimbursement to alternate‐destination transports such as urgent‐care clinics. The official ET3 policy goals emphasize trimming unnecessary ambulance runs, freeing crews for high‐acuity events, and lowering system costs without requiring prior authorization, thereby turning clinical discretion, rather than mileage, into the basis for payment [28]. Experience at the federal level has been more nuanced: in CMS's ET3 model, most treatment‐in‐place encounters were handled through on‐scene tele‐consults and, while short‐term mortality matched that of similar low‐acuity ambulance patients taken to emergency departments, 5‐day hospital‐admission rates were modestly higher; the program's reach was also blunted by COVID‐19‐era start‐up delays, workforce constraints and partner unfamiliarity with the new pathways [29].

Teletrauma has matured from a proof‐of‐concept into a plausible system lever for achieving parity among rural and urban areas, but its adoption trails other telehealth uses and remains uneven. In a national survey of more than 4500 emergency departments, only 8.4% reported using teletrauma in 2020, despite far higher uptake of other telehealth services and despite strong associations with rural location and critical‐access status, which implies that the sites with the greatest need are adopting yet still at low absolute rates [20]. Real‐time trauma consultation can also potentially reduce avoidable transfers, conserve capacity at Levels I and II centers, and accelerate transfers of critical cases when needed, all the while aiming to lower total costs without compromising in‐hospital mortality [30]. Many have argued for the case of teletrauma as a feasible bridge technology for the millions of Americans who lack timely access to Levels I and II expertise, while cautioning that standardized protocols, reimbursement parity, and interoperable data are prerequisites for robust evaluation and scaling these efforts [31, 32]. States can catalyze efforts to implement teletrauma by mandating a sort of hub‐and‐spoke coverage within regional plans, by requiring payer parity of emergency teleconsultation, by supporting interstate clinician practice through participation in compacts and reciprocal licensure, and by integrating teletrauma encounters into statewide trauma registries so that under‐ and over‐triage metrics capture tele‐medical triage as well as physical transfer.

Ensuring a judicious transfer is even more pressing as following the COVID‐19 pandemic, capacity constraints have reduced IHT acceptance in many regions, leaving patients to board in sending EDs and amplifying existing inequities as recent national analyses document declining transfer access and insurance‐linked disparities in transfer and mortality [33, 34, 35]. Including transfer acceptance/denial rates and time‐to‐accept in public reporting, and explicitly counting tele‐consult‐guided “treat in place” decisions in under‐/over‐triage denominators could also help align incentives with the realities of how much capacity there is.

Furthermore, community paramedicine and mobile‐integrated health programs represent a possible pragmatic extension of trauma systems beyond the hospital setting. For example, in a cluster randomized trial of subsidized public housing, the CP@clinic program yielded about 0.90 fewer 9–1–1 calls per month per 100 apartment units, roughly 11 fewer calls per 100 units annually, together with a significant gain in health‐related quality of life and a highly favorable cost–utility profile well below conventional willingness‐to‐pay thresholds (having reduced EMS calls and avoided its associated costs) [36, 37]. In another study, CP@clinic was associated with greater primary care utilization, higher odds of antihypertensive medication initiation, increased receipt of home‐care services, and fewer transitions to long‐term care [38]. Observational and quasi‐experimental evaluations suggest meaningful gains through utilization of such services. While these studies were largely conducted in subsidized housing among older adults, the mechanism of preventing avoidable 9–1–1 calls and ED visits directly frees EMS and ED capacity for time‐critical trauma. It is possible that legislatures could even prioritize mobile‐integrated healthcare‐community paramedic (MIH‐CP) for injury‐adjacent use cases (post‐fall home safety checks, post‐fracture adherence support, wound and suture checks) and require linkage of MIH‐CP encounters to trauma registries and all‐payer claims to quantify downstream effects on transfers, readmissions, and functional recovery. Beyond trauma, MIH‐CP has shown benefits in cardiopulmonary cohorts, suggesting a generalizable capacity effect. On a prospective matched cohort of patients discharged with heart failure, acute myocardial infarction, or chronic obstructive pulmonary disease, a 30‐day community paramedicine home‐visit program was associated with fewer readmissions at 120 and 210 days, fewer emergency department visits over the same period, and lower total costs, with no excess adverse events reported [39, 40].

From a policy standpoint, states can formalize MIH‐CP within trauma system plans, authorize payment for transitional home visits and medications following injury, and require linkage of MIH‐CP encounters to state trauma registries and all‐payer claims datasets so that functional and utilization outcomes are observable beyond simple admission. Given the maturing evidence, legislatures should pair coverage with evaluation, emphasizing high‐risk transitions such as discharge after major orthopedic or neurotrauma, where home‐based risk assessment, falls prevention, and adherence to care plan support are likely to translate into measurable benefits for patients and for reducing EMS workload.

It is often the case that system‐level reconfiguration can improve survival chances. For example, after England established regional Major Trauma Networks, with ambulance bypass to Major Trauma Centers (MTCs), the system saw tangible process gains (more patients treated at MTCs, greater consultant‐led care, faster imaging, and widespread adoption of massive transfusion protocols and tranexamic acid) accompanied by improved outcomes, evidenced by a 19% increase in case‐mix–adjusted odds of survival for severe injury over 2008–2017 and a significant post‐implementation acceleration of benefit of ~0.08 additional survivors per 100 patients each quarter [41, 42]. In Victoria, Australia, the maturation of an inclusive, statewide trauma system with prehospital bypass to Major Trauma Services was associated with substantial risk‐adjusted mortality reductions during 2001–2006, with similar gains in road trauma and serious head injury and contemporaneous improvements in patient‐centered recovery [43]. Furthermore, the regionalization of trauma care improved 12‐month functional outcomes year‐on‐year among blunt major trauma survivors, particularly for those definitively treated at Level I Major Trauma Services [44]. In a pan‐Canadian cohort of 78,807 adults treated at Level I/II trauma centers (2006–2012), risk‐adjusted inpatient mortality fell from 12.1% to 9.9%, an 18.2% relative reduction equating to ~248 additional lives saved in 2012 versus 2006, while mean risk‐adjusted hospital stay shortened from 11.6 to 10.6 days (8.6% reduction; ~10,000 hospital days saved), with significant mortality declines in Ontario, Alberta, and Manitoba and significant LOS reductions in Québec, British Columbia, and Ontario [45]. In the United States, while patient outcomes may be improved for those treated at trauma centers (especially when more available), the absence of a unified national system and persistent rural gaps mean gains have been uneven.

Federal levers can accelerate state action without necessarily needing to preempt it. Congress has periodically reauthorized grant authorities for trauma systems (e.g., the Trauma Systems and Regionalization of Emergency Care programs) and funded military–civilian trauma partnerships via the MISSION ZERO Act, and so reauthorization and full funding through PAHPA/ASPR could possibly condition grants on state adoption of core standards (e.g., data linkage, geriatric triage rules, transfer metrics) [46, 47, 48, 49, 50, 51, 52]. Such conditional grants, akin to other federal–state health compacts, offer a politically palatable path to harmonize some of the minimum standards of care while leaving design flexibility to states.

Public reporting can also be used to catalyze some improvement [53, 54]. Analogous to the ACEP emergency care report cards [55], states (or federal partners via conditional grants) could publish an annual trauma system “report card” with standardized, risk‐adjusted indicators, such as under‐ and over‐triage rates, 90‐min transfer rates for ISS ≥ 15, ED boarding time for admitted trauma patients, tele‐consultation use, and 30‐ to 90‐day functional outcomes. Several states already publish trauma/EMS dashboards and annual reports (e.g., California EMSA statewide EMS/trauma reports, Pennsylvania Trauma Systems Foundation annual reports), demonstrating there is some feasibility to this [56, 57, 58, 59, 60, 61, 62].

In conjunction with these efforts, to address some of the concerns regarding data silos, it is possible that states could consider: (1) mandating NEMSIS v3‐conformant ePCR submission and NTDS‐conformant trauma registry data; (2) standing up a state data trust (with DUAs/MOUs) that performs privacy‐preserving, probabilistic linkage across EMS ePCR, hospital discharge files, trauma registries, APCDs/Medicaid, and IRF‐PAI/rehabilitation datasets; (3) publishing a core trauma system dashboard (under‐/over‐triage, transfer times, ED boarding for admitted trauma, 30‐/90‐day readmission and functional outcomes); and (4) requiring participation as a condition of designation. Several states illustrate pieces of this architecture (e.g., California EMSA's statewide EMS/trauma reporting; Pennsylvania's PTSF annual reporting and funding transparency; state consultation reports that explicitly target under‐/over‐triage and transfer metrics).

## 4 Conclusion

A modern, evidence‐based trauma system requires collaboration at the state and federal levels to address mass‐casualty events stemming from man‐made or natural cause humanitarian disasters. Inter‐state collaboration may also be relevant when considering climate‐related changes—particularly as climate‐amplified mass‐casualty events (wildfires, flooding, severe storms) increasingly cross jurisdictional lines—with a rigorous focus on continuous research to refine its components (Table 2).

**TABLE 2:** Legislative priorities to address trauma system disparities.

Legislative priority	Rationale	Potential policy actions
Unified statewide data systems	Facilitate injury surveillance, EMS/hospital data integration	Mandate comprehensive, consistent data reporting across EMS and hospitals
Geriatric triage standards	Address under‐triage for older adults	Enact legislation requiring geriatric‐specific triage criteria (SBP < 110, GCS ≤ 14)
EMS coverage and resource funding	Mitigate rural hospital closures and staff shortages	Earmark funds for rural EMS training, telemedicine, air transport infrastructure
Regionalized mass casualty preparedness	Improve system‐wide surge capacity	Develop and fund multi‐agency operational plans and telehealth disaster “playbooks”
Rehabilitation and long‐term outcomes	Integrate post‐acute functional recovery as system metric	Require state registries to track functional outcomes and reimburse transitional care

Carefully crafted legislation at the state level, encompassing standardized data capture, geriatric and rural triage adaptations, integrated research networks, and mass casualty readiness, offers a sustainable pathway. Aligning these elements through a health systems approach will be essential to reduce disparities, guide resource allocation, and optimize care across the trauma continuum.

## Funding

The authors have nothing to report.

## Conflicts of Interest

The authors declare no conflicts of interest.

## Data Availability

Data sharing not applicable to this article as no datasets were generated or analyzed during the current study.
